# Rescuing Loading Induced Bone Formation at Senescence

**DOI:** 10.1371/journal.pcbi.1000924

**Published:** 2010-09-09

**Authors:** Sundar Srinivasan, Brandon J. Ausk, Jitendra Prasad, Dewayne Threet, Steven D. Bain, Thomas S. Richardson, Ted S. Gross

**Affiliations:** 1 Department of Orthopedics and Sports Medicine, University of Washington, Seattle, Washington, United States of America; 2 Department of Statistics, University of Washington, Seattle, Washington, United States of America; Stony Brook University, United States of America

## Abstract

The increasing incidence of osteoporosis worldwide requires anabolic treatments that are safe, effective, and, critically, inexpensive given the prevailing overburdened health care systems. While vigorous skeletal loading is anabolic and holds promise, deficits in mechanotransduction accrued with age markedly diminish the efficacy of readily complied, exercise-based strategies to combat osteoporosis in the elderly. Our approach to explore and counteract these age-related deficits was guided by cellular signaling patterns across hierarchical scales and by the insight that cell responses initiated during transient, rare events hold potential to exert high-fidelity control over temporally and spatially distant tissue adaptation. Here, we present an agent-based model of real-time Ca^2+^/NFAT signaling amongst bone cells that fully described periosteal bone formation induced by a wide variety of loading stimuli in young and aged animals. The model predicted age-related pathway alterations underlying the diminished bone formation at senescence, and hence identified critical deficits that were promising targets for therapy. Based upon model predictions, we implemented an in vivo intervention and show for the first time that supplementing mechanical stimuli with low-dose Cyclosporin A can completely rescue loading induced bone formation in the senescent skeleton. These pre-clinical data provide the rationale to consider this approved pharmaceutical alongside mild physical exercise as an inexpensive, yet potent therapy to augment bone mass in the elderly. Our analyses suggested that real-time cellular signaling strongly influences downstream bone adaptation to mechanical stimuli, and quantification of these otherwise inaccessible, transient events in silico yielded a novel intervention with clinical potential.

## Author Summary

Post-menopausal and age-related osteoporosis afflicts large segments of the population and can markedly increase skeletal fragility. Bone fractures that occur as a consequence substantially increase health care expenditures and raise levels of morbidity. While strategies that prevent further loss of bone exist, options that compensate for bone loss accrued over age are less numerous. Physical exercise holds promise in this realm. However, in part due to deficits in how cells within bone respond to skeletal loading, readily complied exercise has proved ineffective in enhancing bone mass in the elderly. In this study, we examined whether the ability of physical exercise to increase bone mass can be restored at advanced age. To this end, we developed a computational model describing how a specific aspect (or pathway) activated in bone cells by skeletal loading may be altered with age. Our model proved successful in describing age-related pathway alterations and identified specific deficits that were amenable to therapeutic manipulation. We subsequently discovered that when an extremely inexpensive, currently approved pharmaceutical is used as a supplement, bone response to skeletal loading was completely rescued in aged animals. We believe that this result provides the rationale to consider this approach as a means to increasing bone mass in the elderly.

## Introduction

Mechanical stimuli are anabolic for bone and hold promise to counteract skeletal fragility associated with bone loss pathologies [1], [2]. Exercise based strategies are especially attractive given the critical need for inexpensive options to treat osteoporosis worldwide [3]. However, readily complied and tolerated exercise has proved ineffective in enhancing bone mass in the elderly population most in need of such interventions [4], [5]. Studies that have examined the apparent ineffectiveness of mild exercise in the elderly using cell culture systems and animal models have sometimes led to conflicting outcomes. For instance, specific aspects of cell signaling pathways may/may not be altered with age [6], [7]. Furthermore, reports suggest that exercise training can elicit enhanced bone tissue responses in animals at advanced age [8], [9]. However, in studies where in vivo deformations and strains induced by mechanical stimuli are equivalently calibrated, observations suggest that aging markedly blunts the osteogenic response to mechanical stimuli [10], [11], and renders adaptation into a low-level binary off-on state [12].

To begin to explore underlying potential for age-related deficits in mechanotransduction function, we first focused upon observations that a single bout of mechanical stimuli (∼100 s) is sufficient to influence bone matrix secretion up to a week later [13]. Furthermore, brief stimuli (∼15 s) repeated every 24 hrs robustly enhances bone formation and bone mass [14]. These observations suggest that cell signaling activated *during* brief stimuli are focal events that guide unique downstream bone adaptation. A variety of second messengers (e.g., Ca^2+^, NO, PGE_2_, cAMP, ATP) are acutely activated by mechanical stimuli. Of these, the Ca^2+^ ion second messenger system may be unique in its specificity given that all important aspects of mechanical stimuli that influence bone formation in vivo (e.g., magnitude, strain rate, frequency, rest intervals) have been observed to provoke highly specific real-time Ca^2+^ oscillations in bone cells in vitro [7], [15]–[17]. Furthermore, while blockade of Ca^2+^ signaling disrupts mechano-responsive gene expression [18], [19], gap junctional communication integrates Ca^2+^ signaling within the bone cell network [20] and may be required to influence ‘effector’ osteoblast cell differentiation [21], [22]. Downstream of signaling through the Ca^2+^ ion system, activation of a variety of transcription factors (e.g., NF-κB, JNK, NFAT) provides putative links between cell responses on the order of seconds to cell function over successive days of the week. Of these, the nuclear factor of activated T-cell family of transcription factors (NFAT c1–c4) may be unique. Specifically, NFAT activation dynamics (within minutes) is remarkably specific to Ca^2+^ amplitudes and frequencies known to influence distinct downstream cell functions, including proliferation, differentiation and apoptosis [23], [24]. Taken together, and given recent evidence of NFAT's critical involvement in bone mechanotransduction [25], [26], characterization of Ca^2+^/NFAT signaling and age-related alterations in bone cells in vivo may prove useful. However, it is not currently possible to experimentally quantify real-time activation of Ca^2+^/NFAT signaling in situ within bone.

**Figure 1:**
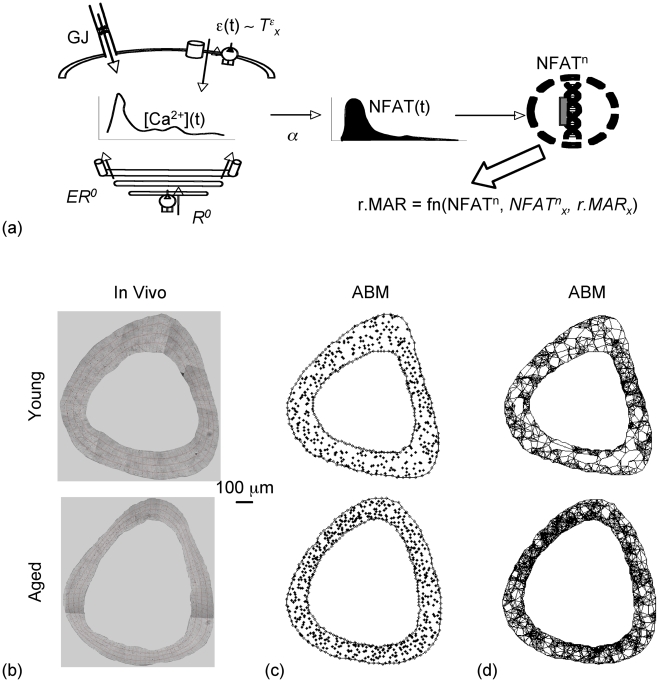
ABM for Ca^2+^/NFAT signaling in bone cells networks and relation to relative mineral apposition rates induced in osteoblasts. In the model (a), mechanical strain (ε(t)) at the location of the osteocytic cell induced an influx of Ca^2+^ ions (via a relation between ε(t) and model parameter T^ε^_x_, the threshold strain magnitude at which maximal Ca^2+^ oscillation amplitudes are induced). Additional Ca^2+^ influx was modeled to occur via gap-junctional (GJ) influx of Ca^2+^ ions from physically connected neighboring cells (a). The filling state of the ER (that was replenished at a parameterized maximal rate, R^0^) and the influx-induced release of Ca^2+^ ions from the ER (with a parameterized maximal store capacity, ER^0^) modulated the Ca^2+^ oscillations that arose at any given time within osteocytes and precursor cells (a). In precursor cells, NFAT dephosphorylation was influenced by prior Ca^2+^ oscillation histories (via a relation controlled by the parameter, α). Dephosphorylated NFAT translocated to the nucleus and upon DNA binding, controlled relative mineral apposition rates in differentiated osteoblasts (via a relationship defined by parameters NFAT^n^_x_, the maximal NFAT DNA binding capacity, and r.MAR_x_, the maximal relative mineral apposition rate, a). To implement the model in idealized representations of bone cell networks in situ, thin cross-sections from the mid-shaft tibiae of young (4 Mo; top panel, b) and senescent female C57BL/6 mice (22 Mo; bottom panel, b) were imaged. Osteocyte (‘asterisk’) and surface precursor cell (‘diamond’) locations within the cortex were determined from imaging and specified for model representation of cell networks in young (top panel, c) and aged animals (bottom panel, c). Finally, based upon literature, cell-cell functional connections were specified for young (top panel, d) and aged models (bottom panel, d).

Given this inaccessibility, we previously developed a model for Ca^2+^ signaling induced in simple networks of osteocytic cells subject to mechanical stimuli using a numerical/computational technique called agent-based modeling (ABM) [27]. ABMs have been used to explore bottom-up emergent phenomena in biological systems ranging from cell-cell interactions [28] to ecosystem dynamics [29]. In our prior study, we used the characteristic of the ABM technique to examine how local properties (e.g., cell response thresholds) and dynamic interactions between local properties over time (e.g., cell-cell communication) influenced the emergence of global properties (e.g., collective Ca^2+^ signaling in cell ensembles). Based in part upon our original framework [27], here we present an ABM of Ca^2+^/NFAT signaling *in situ* within bone's cellular syncytium and its relation to osteoblastic relative mineral apposition rates induced by mechanical loading (r.MAR; where r.MAR = MAR in loaded−MAR in contralateral bones; Fig 1). We hypothesized that the Ca^2+^/NFAT ABM would accurately simulate bone adaptation, predict aspects of the modeled pathway that were altered by aging and identify aspects amenable to intervention.

To test our hypothesis, we examined whether the model could be calibrated to accurately simulate relative periosteal bone formation rates (rp.BFR) induced in vivo by a variety of mechanical stimuli in young and senescent female C57BL/6 mice [12], [30]. We subsequently used the model to predict age-related deficits within the pathway. Similar to the literature, the model predicted age-related deficits in the ability of Ca^2+^ oscillations to dephosphorylate NFAT [31] and in NFAT DNA binding capacity [32], as factors underlying the muted adaptation observed in senescent animals [12]. We then surveyed the literature to examine pharmaceutical agents that could modulate these deficits and identified Cyclosporin A (CsA) as one possibility [33]–[35]. Lastly, to validate model predictions, we examined whether the use of low-dose CsA as a supplement could restore the bone formation response to loading when implemented in vivo in senescent animals.

## Results

**Figure 2:**
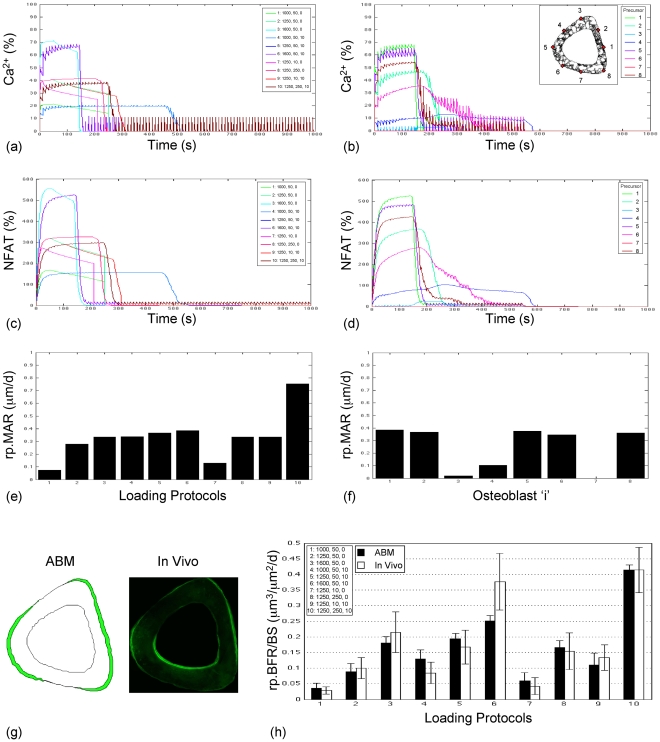
The ABM accurately simulates rp.BFR (mean + s.e.) induced by loading in young adult animals. At the young optima vector (Table 2), unique Ca^2+^ oscillations were induced by each loading protocol in surface cells (a; fluctuations in precursor ‘1’ is indicated; cell position indicated in the inset of figure b) and by a given protocol in surface precursors around the bone surface (b; fluctuations for protocol ‘6’ is indicated). The resulting NFAT dephosphorylation dynamics represented a ‘memory’ of Ca^2+^ oscillation histories across loading protocols (c; in precursor ‘1’) and around the bone cortex (d; for protocol ‘6’). As a consequence of activation of Ca^2+^/NFAT signaling within the bone cell syncytium, unique rp.MAR was induced in differentiated periosteal surface osteoblasts by each protocol (e; for osteoblast differentiated from precursor ‘1’) and in osteoblasts around the bone's surface (f; for protocol ‘6’; where locations of differentiated osteoblasts are the same as their ‘parent’ precursor cells provided in the inset of b). The resulting locations of bone formation were qualitatively similar to that observed in vivo (g; for protocol 6). Finally, ABM simulation of rp.BFR induced at the tissue level by 10-independent protocols was not significantly different from that measured in vivo in young adult animals (h; mean ± s.e., p = 0.67). Note that protocols are denoted as induced strains, number of load cycles/d and the rest interval (seconds) inserted between each load cycle (e.g., 1250, 250, 10 would denote a loading protocol that induced 1250 με peak strain over 250 cycles/d, with a 10 s rest interval inserted between each load cycle). Also note that in (a) and (c), responses to protocol 5 were induced over a shorter duration and overwritten by response to protocol 10.

**Figure 3:**
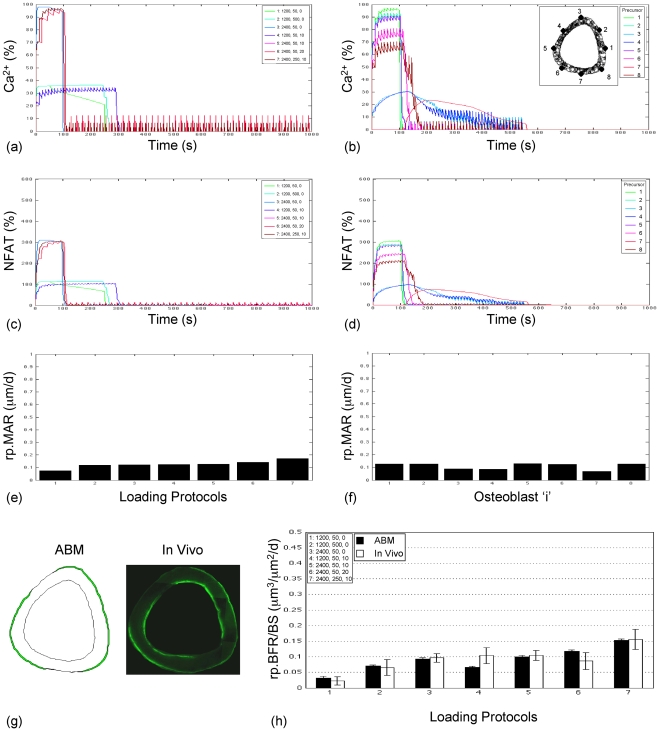
The ABM accurately simulates rp.BFR (mean + s.e.) induced by loading in senescent animals. Intracellular Ca^2+^ oscillations induced by each loading protocol in surface cells over 1000 seconds (a; fluctuations in precursor ‘1’ is indicated; cell position indicated in the inset of figure b) and by any given protocol in surface precursors around the bone surface (b; fluctuations for protocol ‘5’ is indicated) at the aged optima vector (Table 2). The resulting NFAT dephosphorylation dynamics across loading protocols (c; in precursor ‘1’) and around the bone cortex (d; for protocol ‘5’) were qualitatively muted compared to the case in young ABM simulations (Fig 2). The consequent lowered rp.MAR was induced in surface osteoblasts by each protocol (e; for osteoblast ‘1’) and in osteoblasts around the bone's surface (f; for protocol ‘5’; where locations of differentiated osteoblasts are the same as their ‘parent’ precursor cells provided in the inset of b), but where the resulting bone formation was more extensively distributed around the cortex vs that in young (g; for protocol 5). Finally, ABM simulation of rp.BFR induced at the tissue level by 7-independent protocols was not significantly different from that measured in vivo in senescent animals (h; mean ± s.e., p = 0.57). Note that in (a) and (c), responses to protocol 5 were induced over a shorter duration and overwritten by response to protocol 7.

The ABM of the Ca^2+^/NFAT pathway incorporated sufficient parametric complexity to simulate rp.BFR induced by a variety of loading protocols in young adult [30] and senescent animals [12]. We identified an optimal ABM parameter vector by maximizing likelihoods via an optimization procedure, called simulated annealing that explored the model parameter space (Table 1). At the young and aged maximum likelihood estimate (MLE, Table 2), simulation of Ca^2+^ oscillations in individual cells (Fig 2, 3) was qualitatively similar to that observed in in vitro systems [15], [17]. Further, NFAT signaling downstream of Ca^2+^ oscillations conferred a high-fidelity memory, and the resulting relative mineral apposition rates in individual cells retained information regarding the distinct osteogenic potentials of the loading protocols (Fig 2, 3). Importantly, the consequent tissue level rp.BFR simulated for animal specific strains was not significantly different from in vivo data in young adult and in senescent animals (p = 0.67, 0.57 respectively; Fig 2, 3).

**Table 1:** Range of parameter values.

	*T^ε^_x_* (με)	*ER^0^* (%)	*R^0^* (% s^−1^)	*α*	*NFAT^n^_x_* (%)	*r.MAR_x_* (µm/d)
Min	0	0	0	0.0	0	0.0
Max	5,500	50,000	200	1.0	10,000,000	50.0

Ranges utilized in estimating the 6 independent ABM parameters.

**Table 2:** Young (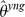) and aged (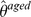) MLEs and 95% confidence intervals.

	‘Young’			‘Aged’		
	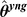	Lower	Upper	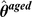	Lower	Upper
T^ε^_x_ (με)	**2,524**	1,571	3,517	**2,619**	1,516	4,608
ER^0^ (%)	**9,430**	5,115	19,561	**9,215**	4,059	21,815
R^0^ (% s^−1^)	**1.79**	0.088	5.78	**1.03**	0.0	10.6
α	**0.873**	0.084	0.99	**0.684^#^**	0.0	0.99
NFAT^n^_x_ (%)	**239,610**	4,783	1.388E6	**130,550^#^**	19,766	5.259E6
r.MAR_x_ (µm/d)	**2.58**	0.43	12.63	**2.03**	0.24	9.76

Optimal ABM parameter vectors (‘Young’ and ‘Aged’ MLEs, with 95% confidence intervals) that enable accurate simulation of rp.BFR data from young adult and senescent animals [12], [30], respectively. The optima (MLE) was estimated by maximizing likelihoods and involved simulating ∼2×10^9^ cell ‘states’ per likelihood function evaluation, and required ∼5×10^5^ function evaluations to determine the MLE, and ∼3×10^7^ function evaluations to determine the confidence intervals (at 0.6 CPU seconds/function evaluation). Parameters significantly altered by aging are noted (#).

**Figure 4:**
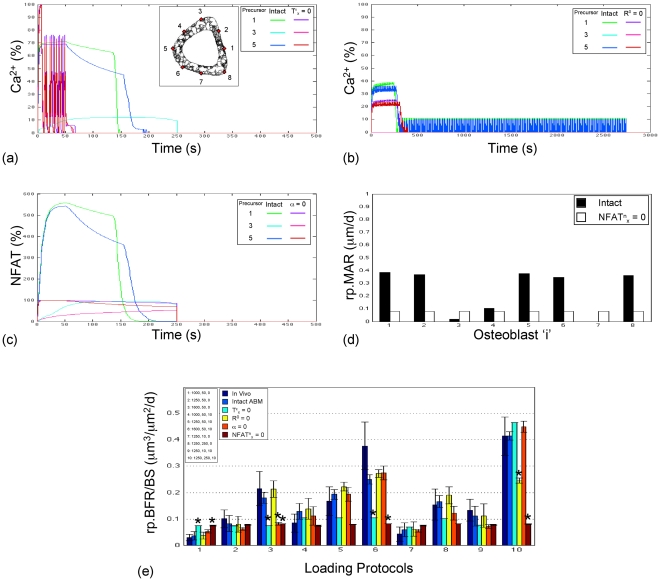
Synthetic knockout of sub-critical components of the Ca^2+^/NFAT pathway significantly degrades model accuracy in simulating loading induced bone formation data in young adult animals. Knockout of parameter T^ε^_x_ substantially altered intracellular Ca^2+^ oscillations (a; illustrated in select surface precursors for protocol ‘3’; oscillations induced by parameter ‘knock-out’ vs by ‘intact’ ABM simulations). Knockout of parameter R^0^ eliminated secondary transients in intracellular Ca^2+^ oscillations (b; illustrated in select surface precursors for protocol ‘10’). Knockout of parameter α diminished Ca^2+^ oscillation induced dephosphorylation of NFAT protein (c; illustrated in select surface precursors for protocol ‘3’). Knockout of parameter NFAT^n^_x_ substantially altered and rendered binary (i.e., on/off) downstream rp.MAR induced in surface osteoblasts (d; illustrated in select surface osteoblasts for protocol ‘6’; where locations of differentiated osteoblasts are the same as their ‘parent’ precursor cells provided in the inset of a). Consequently, knockout of each of these components of the Ca^2+^/NFAT pathway significantly altered tissue level rp.BFR in a protocol specific manner (e; mean ± s.e., ‘*’ indicates p<0.05 vs in vivo).

To test for redundant model parameters, we next examined whether the ABM would remain ‘true’ upon further simplification via parameter ‘knock-outs’ (i.e., constraining parameter values = 0, one at a time). ‘Knock-out’ of parameters resulted in simulations that were significantly different from both the experimental rp.BFR data (p≤0.02; Table S1) [12], [30] and from rp.BFR simulated by the unconstrained ABM (p≤0.01; Fig 4). Of note, removing parameters ER^0^ or r.MAR_x_ always resulted in rp.BFR_i_ = 0 (regardless of the loading protocol ‘i’ and whether the remaining parameters were optimized). Post-hoc analysis of ABM simulations suggested that ‘knock-out’ of the remaining parameters de-couples Ca^2+^ oscillation from induced strains (T^ε^_x_ = 0), inhibits secondary Ca^2+^ transients (R^0^ = 0), decreases Ca^2+^ induced dephosphorylation of NFAT (α = 0) and renders mineral apposition rates into a binary ‘on/off’ state in osteoblasts (NFAT^n^_x_ = 0; Fig 4). As such, attempts to simplify the model via functional ‘knock-outs’ significantly degraded model ability to simulate the in vivo data (Fig 4).

The ability of the unconstrained ABM to accurately simulate the in vivo data from both young [30] and senescent animals [12] uniquely permitted an examination of aging related alterations in the model's parameters. The null-hypothesis that aging does not alter the Ca^2+^/NFAT pathway ABM was rejected (6-df, p<0.0001). Given this result and in the context of literature reports, analysis of null hypotheses that aging does not alter the parameters T^ε^_x_, ER^0^, r.MAR_x_ were accepted both when considered individually (p = 0.31, 0.58 and 0.75, respectively; 1-df) and synchronously (p = 0.85; 3-df). Subsequently, when it was assumed that aging does not alter T^ε^_x_, ER^0^, and r.MAR_x_, the null hypothesis that aging does not degrade components (1-df) was rejected for the parameter α (p = 0.004) and NFAT^n^_x_ (p = 0.002) but accepted for the parameter R^0^ (p = 0.27). Lastly, we examined the influence of simulated interventions that restored (the identified) age-related deficits in components of the Ca^2+^/NFAT pathway. We found that synchronously restoring parameters with significant age-related deficits (i.e., α, NFAT^n^_x_) to their young optima values significantly increased rp.BFR induced by a variety of loading stimuli (p<0.001; mean: +95%, range: +49% to +120%).

**Figure 5:**
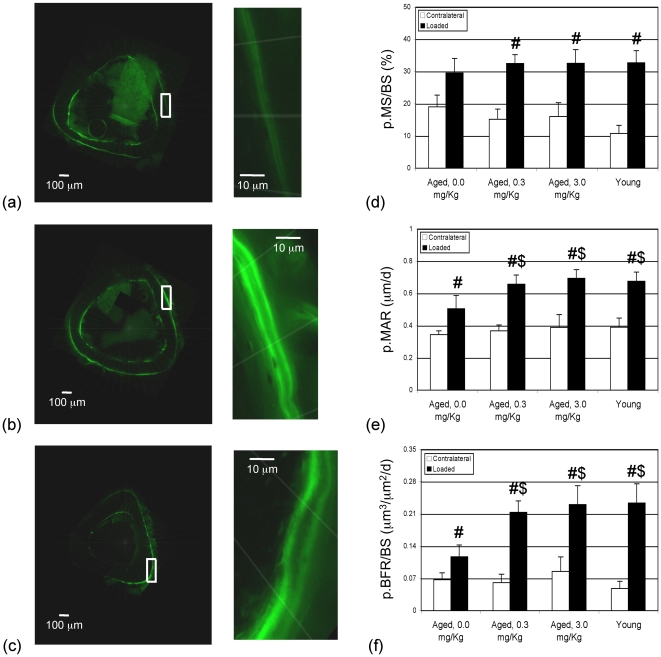
Mechanical loading supplemented with low-dose CsA restored bone formation response at senescence to levels observed in young. Illustrative florescent images at the mid-shaft of loaded tibia from senescent mice (22 Mo) that were vehicle treated (a; 0.0 mg/kg), supplemented with CsA (b; 3.0 mg/kg), and from young mice (c; 4 Mo). Images in the mid-panels represent insets magnified. Periosteal mineralizing surface (p.MS/BS; d), periosteal mineral apposition rate (p.MAR; e) and periosteal bone formation rates (p.BFR/BS; f) in senescent mice that were vehicle treated (0.0 mg/kg), treated with CsA at two dosages (0.3, 3.0 mg/kg), or in young animals that received loading calibrated to induce equivalent periosteal strains. Data represent mean+s.e.; # significantly different vs contralateral bones, $ vs loaded limbs of vehicle treated animals, at p<0.05.

We next performed in vivo experiments to validate model insights and to thereby evaluate the promise of this strategy (i.e., interventions that could restore age-related deficits in α, and NFAT^n^_x_). Specifically, senescent female C57BL/6 mice (22 Mo) were subjected to mechanical loading 3 d/wk for 3-wks with/without low-dose CsA supplements (0.3 or 3.0 mg/kg s.c.). Additionally, to examine the extent to which bone adaptation can be rescued at senescence, young female C57BL/6 mice underwent an identical loading protocol (without CsA supplementation). We found that in contralateral bones (not subject to exogenous loading), periosteal mineralizing surface (p.MS/BS, p = 0.29), mineral apposition rate (p.MAR; p = 0.82) and bone formation rate (p.BFR/BS; p = 0.56) were not significantly different between aged animals with or without CsA supplements or compared to young animals (Fig 5). In these experiments, loading induced periosteal strains were not significantly different between senescent and young mice (p = 0.27). While p.MS/BS was not significantly different in the loaded limbs across groups, we found that loading induced p.MAR (p<0.01) and p.BFR/BS (p = 0.04) were significantly lower in vehicle treated senescent mice compared with young mice (Fig 5). In contrast, loading supplemented with CsA at both 0.3 and 3.0 mg/kg significantly enhanced both p.MAR (p<0.01 for both dosages) and p.BFR/BS (p<0.01 for both dosages) compared to that in vehicle treated senescent mice and to levels not different from that in young animals (Fig 5, p>0.55).

**Figure 6:**
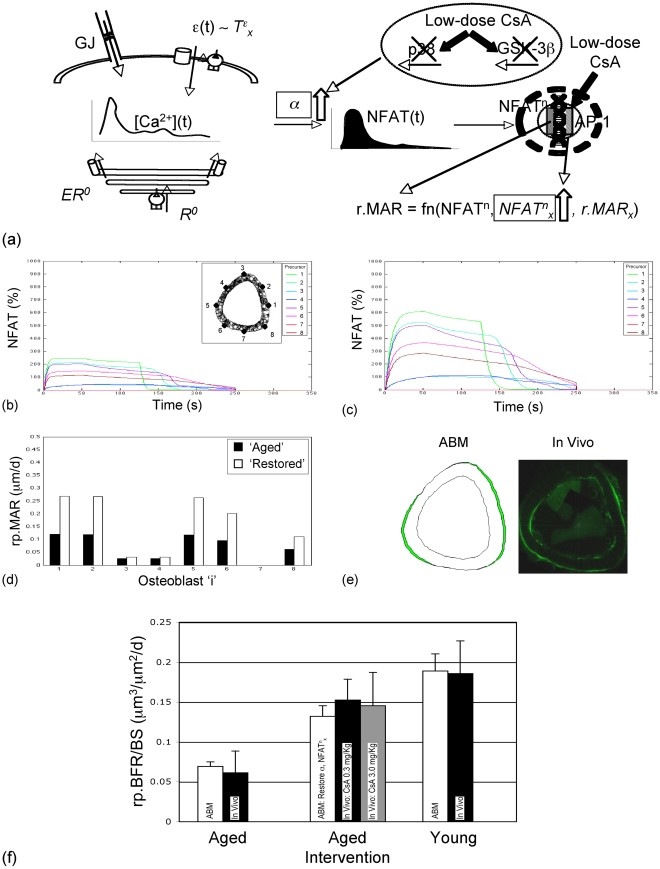
Simulated restoration of age-related deficits in the Ca^2+^/NFAT pathway predicts loading induced rp.BFR in senescent mice supplemented with CsA. Hypothesized mechanisms of action of low-dose CsA in restoring age-related deficits in model parameters α and NFAT^n^_x_ (a). Compared to the aged model simulations (b), restoring age-related deficits in model parameters increased NFAT dephosphorylation and signaling in precursor cells around the bone cortex (c; cell locations are indicated in the inset of b), and consequently increased rp.MAR in differentiated osteoblasts (d; where locations of differentiated osteoblasts are the same as their ‘parent’ precursor cells provided in the inset of b). Post-restoration of age-related deficits, model simulation of resulting bone formation was qualitatively similar to that induced by loading supplemented with CsA in vivo (e). Finally, in the context of animal specific loading induced strains, model simulations of rp.BFR at the tissue level was not significantly different from in vivo data in aged and young animals subject to mechanical loading (50 c/d, 1700 με, 1-Hz; f). Importantly, model simulations of tissue level rp.BFR at senescence, post restoration of α and NFAT^n^_x_ to their young values (Table 2), was not significantly different from in vivo data in senescent animals subject to loading supplemented with CsA at either of the defined dosages (f).

Lastly, we examined whether the model could be used to predict rp.BFR induced in senescent animals supplemented with low-dose CsA. Specifically, to simulate the hypothesized mode of interaction between loading and low-dose CsA (Fig 6 a), model parameters significantly degraded by age (i.e., α, NFAT^n^_x_) were synchronously restored to their young optima values (Table 2). Simulated restoration of age-related deficits in α and NFAT^n^_x_ (that are downstream of Ca^2+^ signaling) did not differentially influence Ca^2+^ oscillations in cells around the surface in response to the loading protocol (1700 με, 50 c/d; not shown here for brevity). Restoring α and NFAT^n^_x_ resulted in increases in NFAT dephosphorylation in surface precursors (Fig 6 b, c) and consequent r.MAR (Fig 6 d) in differentiated osteoblasts around the bone surface compared with the case with aged model parameters (Table 2). Validating model predictions, we found that simulation of rp.BFR was not significantly different from in vivo data (Fig 5 f) obtained from senescent mice subject to loading without or with CsA, and from young mice subject to loading (p = 0.87; Fig 6 f).

## Discussion

We present the development of an ABM that describes activation of Ca^2+^/NFAT signaling within and between cells in bone. The model incorporated sufficient complexity (i.e., parameters and analytics) to accurately simulate relative periosteal bone formation rates induced by a variety of mechanical stimuli in young adult and senescent animals. Subsequent in silico ‘knock-out’ of ABM parameters indicated that attempts to further simplify the model significantly degraded the ability to simulate the in vivo data. Model predictions of age-related alterations (or lack thereof) in 5 of 6 ABM parameters were similar in direction to that reported individually in the literature. Model simulations suggested that restoring deficits in NFAT dephosphorylation/translocation and DNA binding capacity could substantially enhance bone's responsiveness to loading at senescence. Finally, follow-up in vivo experiments validated the model predictions and confirmed that low-dose CsA, when used as an adjuvant to skeletal loading, can completely rescue loading induced bone formation in senescent mice.

The broad assumption underlying our study is that activation of intracellular Ca^2+^ oscillations and subsequent activation of the NFAT pathway is a critical mechanism underlying bone mechanotransduction. As described in the introduction, the literature clearly supports this hypothesis [23]–[26]. Our model represents a first attempt at quantifying activation of the Ca^2+^/NFAT pathway in situ and its downstream consequences for bone tissue. However, given that a number of additional second messenger systems are also activated by brief mechanical stimuli (e.g., cAMP, NO), and that transcription factors in addition to NFAT also modulate and influence cell and tissue adaptation downstream [1], [36], [37], we expect to explore these interacting pathways in future model refinements.

Another limitation was associated with the mathematical formulations used to simulate loading induced activation of the Ca^2+^/NFAT pathway. The formulations were specified to efficiently (computationally) describe experimental observations and were similar to previous mathematical descriptions [17], [21]–[23], [27], [37]–[45]. While the current model parameterization undoubtedly represents a simplified description, the ABM technique would readily enable finer mathematical specifications of more fundamental mechanisms and processes via which Ca^2+^/NFAT signaling emerges in individual cells. One direct limitation of the ABM formulation is that model time increments are restricted to 1 s and greater (and hence, the current model cannot simulate adaptation induced by aspects of loading such as strain rates and frequencies) [2], [46]. While formulating the biological observations in a more refined manner (e.g., differential equation based) would address this limitation, such an approach would also be expected to substantially increase the computational effort required. Also, the parameter optima for this ABM (Table 2) are relevant only to this current model. As such, any further refinement of the model would be likely to result in new optima. In the context of these observations, the accuracy of our model predictions (age-related pathway deficits, prediction of the effects of targeted interventions) does lend initial validity for the implemented formulations.

We next consider some of other simplifications and limitations of the model. For instance, while the current model accurately simulates adaptation at the periosteal surface, it does not accurately predict adaptation at the endocortical surface. This is not entirely surprising given that the cells responsible for endocortical adaptation may be unique from periosteal precursors, and their distinct milieu can induce differential responses to mechanical stimuli [47]. Further, cells present at the endocortical surface may receive stimulation secondary to loading in a very different manner (e.g., marrow pressurization [48]) compared to periosteal cells - a difference that may instead contribute to the differential response at the bone surfaces. As such, planned model expansions to simulate adaptation at the endocortical surface will have to address some of these possibilities. Another limitation is that the current model is a 2-D description of a biological process that undoubtedly unfolds in 3-D. While there is no feature within the mathematical model per se that precludes extension to 3-D, a representation of the cell network in the tibia diaphysis via imaging is first needed to incorporate 3-D network topology and anatomy within the model, and will be a focus of future model refinements. A third limitation is that the model does not explicitly incorporate parameters that distinguish between the different loading protocols in previous young (3 days/wk for 3-wks) vs senescent animal experiments (5 days/wk for 2-wks) [12], [30] from our group that were used in model calibration. The reasons for this omission were: 1) reports, including our preliminary data, which suggest that loading bone every 24 hrs vs 48 hrs does not differentially influence bone formation [49], 2) the time between fluorochrome labeling were the same in the two studies (9 days) [12], [30], 3) the current model can accurately simulate the previous data [12], [30] without an additional parameter, and 4) bone formation rates induced in our current in vivo experiments and model predictions in aged mice (1700 με, 50 c/d, 3 days/wk, 3-wks; Fig 6) were per expectations given our previous data in senescent mice (that underwent 50 cycles/d of loading for 5 days/wk over 2-wk protocols at both 1200 με and 2400 με). However, the lack of explicit model consideration of this loading bout scheduling aspect of protocols is a limitation that will need to be addressed if broader optimization questions are to be examined (e.g., when and how often is it best to load bone?).

Despite these and other limitations, analysis indicated that the model of the Ca^2+^/NFAT pathway was *sufficient* to simulate bone formation induced in both young adult and senescent animals in response to a wide variety of loading stimuli [12], [30]. In the statistical realm, refinement of our idealized model for Ca^2+^/NFAT signaling, parametric inclusion of other second messengers (e.g., NO, ATP) and interacting pathways (e.g., MAPK, Wnt signaling) [33], [50], or alternate mathematical forms will not result in significantly more accurate simulations of the data at hand. As such, we believe that this result establishes the current model as a critical first step in our exploration of bone mechanotransduction in silico. Furthermore, given the modularity of the ABM technique, our approach readily permits future ABM expansions that address limitations inherent to the current model. We expect that such iterative expansions could lead to more comprehensive models that provide increasingly nuanced representations of mechanotransduction while conferring the ability to predict and optimize bone adaptation induced by a substantially wider variety of mechanical stimuli.

On the other hand, attempts to simplify the current model of the Ca^2+^/NFAT pathway via parameter ‘knock-outs’ significantly degraded the accuracy of simulations. Similar to literature reports [40], knock out of the parameter specifying the maximal capacity of ER Ca^2+^ stores (ER^0^ = 0) abolished intracellular Ca^2+^ oscillations and eliminated all further downstream outcomes. Knock-out of the parameter specifying the maximal rate of osteoblastic mineral apposition (r.MAR_x_ = 0) represented the trivial case where rp.BFR was always zero. As the ABM was unable to adapt to these knock-outs (via optimization of the remaining parameters), the parameters ER^0^ and r.MAR_x_ were therefore critical components of our model of the Ca^2+^/NFAT pathway. While knock-outs of the remaining parameters (T^ε^_x_, R^0^, α and NFAT^n^_x_) were not synthetically lethal for bone formation (i.e., rp.BFR ≠ 0), they nevertheless caused significant loading protocol specific declines in the accuracy of ABM simulations of the in vivo data (Fig 4). This was despite the flexibility of the model and the ability of the remaining intact parameters to adapt (via optimization and location of alternate MLEs under the knockout constraint). Taken together, these results further suggest that while the 6-parameter baseline model was sufficient and accurate, further simplification of the model (via parameter ‘knock-outs’) significantly degraded model ability to simulate the data at hand [12], [30].

The highly specific adaptation that resulted from simulated knock-out of ‘sub-critical’ components of the pathway had an alternate utility as they further elucidated the functioning of the pathway (as modeled). Specifically, knock-out of parameter T^ε^_x_ decoupled strain amplitudes from the Ca^2+^ dynamics and caused highly unusual Ca^2+^ oscillations in cells (Fig 4 a). While these unusual Ca^2+^ oscillations occurred directly as a consequence of compensatory adaptation of remaining ABM parameters, it is unclear whether they are representative of what might occur in vivo (or in vitro) should such a knock out prove feasible. Ultimately, knockout of parameter T^ε^_x_ resulted in significant differences in simulated rp.BFR in loading protocols where increasing strains induce differential adaptive responses. Knock-out of the parameter R^0^ prevented re-filling of stores, eliminated secondary intracellular Ca^2+^ transients, and despite model compensation, significantly degraded rp.BFR simulated when bones were subject to the extended rest-inserted loading protocol (that was most impacted by the lack of secondary Ca^2+^ transients). Knock-out of the parameter α decoupled the ‘memory’ conferred to NFAT dephosphorylation events from upstream Ca^2+^ oscillation histories, and impacted the simulated rp.BFR responses to cyclic loading stimuli (the secondary Ca^2+^ transients induced by rest-inserted loading shielded this stimulus from the effects of knocking out parameter α). However, the effect of knocking out parameter NFAT^n^_x_ was more ubiquitous and significantly altered simulated rp.BFR for both cyclic and rest-inserted loading protocols. Of note, experimental exploration of some of these knock-outs is possible in vivo and could form the basis for additional, future model validation. For example, knock-out of model parameter R^0^ could be partially achieved via functional knock-out of proteins regulating SERCA pumps [51]. Additionally, knock-out of the parameter α could be achieved, for instance, by modulating expression of negative feedback elements of the Ca^2+^/NFAT pathway such as RCAN1 [52]. Each of these functional knockout experimental models could provide extremely valuable tools to further validate the ABM, and more importantly, to investigate bone mechanotransduction function in finer detail.

The ability to accurately simulate data in both young adult and senescent animals (without resorting to additional qualifiers) enabled us to examine age-related alterations in our model for the Ca^2+^/NFAT pathway. Of note, parameter alterations were not specified a priori. Similar to literature, analysis suggested that aging does not significantly alter three components of the modeled pathway: 1) the induced strain magnitude that maximizes Ca^2+^ amplitudes (T^ε^_x_) [7], 2) the Ca^2+^ sequestration capacity of the ER stores (ER^0^) [53], and 3) the maximal rates of mineral apposition by osteoblasts (r.MAR_x_) [54]. Subsequent analysis predicted that aging significantly decreased Ca^2+^ induced dephosphorylation/translocation of NFAT (α) [31], and decreased NFAT DNA binding capacity (NFAT^n^_x_) [32]. However, contrary to literature [55], predicted alterations in Ca^2+^ store refilling rates (R^0^) did not attain statistical significance. It is possible that addressing model limitations (e.g., addressing our lack of consideration of cell viability at senescence [56]) could resolve this one apparent inconsistency. However, it also remains to be determined whether the reported deficit in R^0^ occurs synchronously with correctly predicted age-related deficits (i.e., in α, NFAT^n^_x_) [31], [32] and lack of deficits in other parameters (i.e., in T^ε^_x_, ER^0^, r.MAR_x_) [7], [53], [54], and in the specific context of bone response to mechanical stimuli. Taken together, we believe that the validity of the model is augmented by its ability to correctly predict the direction of age-related alterations in 5 of 6 components of the modeled Ca^2+^/NFAT pathway.

By describing pathway alterations and their relation to bone adaptation within a consolidated framework, the ABM provided a unique tool to query the impact of targeted interventions. Interestingly, a hypothetical simulated intervention that fully and synchronously restored parameters significantly degraded by aging (α, NFAT^n^_x_) to their young ‘states’ was found to nearly double bone formation response to loading at senescence. To explore and exploit this promising prediction, we considered the use of low-dose CsA supplements to mechanical stimuli. CsA is a currently approved immunosuppressant normally regarded as an inhibitor of NFAT signaling. There is substantial conflict regarding the influence of CsA upon bone adaptation, with reported effects dependent upon a number of cofactors, including experimental system and dosage [57], [58]. A recent study clarifies these inconsistencies [35], and suggests that CsA has bi-phasic effects in the trabecular compartment of bone in young adult mice (anabolic at low dosages and inhibitory at high dosages). However, to our knowledge, no previous study has examined whether and how CsA and exogenous mechanical stimuli might interact and modulate bone adaptation. Here, we hypothesized that supplementation with low-dose CsA would mitigate predicted deficits in loading (and Ca^2+^) induced dephosphorylation and nuclear translocation of NFAT (α) in part via CsA suppression of negative regulators such as p38 [34] and/or GSK-3β [33] (Fig 6 a). We additionally speculated that CsA supplementation could also effectively counteract predicted deficits in NFAT-DNA binding (NFAT^n^_x_) by enhancing cooperative binding interactions between loading induced translocation of NFAT and CsA enhanced activation of transcription factor families such as AP-1 [35] (Fig 6 a).

We found that supplementation with CsA (at both 0.3 and 3.0 mg/kg) significantly enhanced periosteal mineral apposition rates (by over 50%) and bone formation rates (by over 80%) compared to that induced by loading alone, and to levels not different from that observed in young animals. While the mechanisms underlying this interaction are not known at this time, we expect that a combination of RT-PCR, immunohistochemistry (for NFAT proteins and targets of the pathway such as RCAN1), and primary culture in vitro experiments might elucidate how CsA and loading interact in the aged skeleton. In this context, while the agreement between model simulations and experimental data in senescent mice supplemented with CsA validates model predictions (Fig 6), it also provides the rationale for initially investigating CsA-loading interactions as hypothesized (Fig 6a). Importantly, the low-dose CsA supplements tested here were observed to completely rescue loading induced bone formation in senescent animals, a deficit that, to our knowledge, has not previously been overcome. These pre-clinical data suggest that CsA in combination with readily complied, mild physical exercise could represent an extremely low-cost anabolic option to augment bone mass. Specifically, we estimate that low-dose CsA could be utilized as an adjunct with mild physical activity for less than $1 US/month [59]. If optimized, and efficacy is borne out in clinical trials, this intervention would enable an inexpensive, anabolic treatment option that could be readily implemented in elderly populations at risk in developed and developing countries alike.

In summary, we present an agent-based model that describes, for the first time, how bone tissue formation in response to brief loading might be controlled by real-time signaling interactions within the bone cell syncytium. Despite the relative simplicity of our approach compared to complexity of bone mechanotransduction, the model was sufficiently sophisticated to enable successful calibration and hence, was able to quantitatively simulate bone formation rates induced by a variety of mechanical stimuli in both young adult and senescent animals. Post calibration, the model was found to correctly predict the directions of age-related alterations in 5 of 6 modeled components of the Ca^2+^/NFAT pathway and identified deficits that were amenable to therapeutic manipulation. Validating model predictions, supplementing mild mechanical stimuli with low-dose CsA was observed to completely restore the bone formation response to loading at senescence. Our model-enabled discovery provides the direct rationale to consider this currently approved pharmaceutical alongside mild physical exercise as an inexpensive, yet potent therapy to augment bone mass in the elderly. These results also further emphasize that intracellular events initiated during transient/infrequent stimuli (and deficits within) may have profound and lasting influences upon cells, tissues, and organs. Given the inaccessibility of such real-time signaling pathways in biological systems and the success of an agent-based modeling approach in this context, we speculate that an analogous approach may prove successful in understanding and predicting the behavior of other primarily homeodynamic systems facing sudden perturbations (e.g., adaptation following musculoskeletal trauma).

## Methods

In the following, we first describe our idealized representation of bone anatomy and cell network topology. Second, we describe the loading induced strain environment at cell locations within the murine tibia mid-shaft. Third, we describe our development of a parametric ABM of the Ca^2+^/NFAT pathway and its relation to mineral apposition rates in osteoblasts. Fourth, we describe the methods and statistical approaches used to determine model parameters and to analyze ABM simulations. Lastly, we describe our in vivo experiments in senescent animals that served to validate model simulations.

### Bone anatomy and cell network topology

The ABM was developed to examine signaling interactions between cells at the murine tibia mid-shaft, the site where experimental data had previously been measured (Table S1) [12], [30]. To determine cell network topology at the tibia mid-shaft (Fig 1), we obtained thin (5 µm) decalcified sections from the tibia mid-shaft of young adult (4 Mo, n = 6) and senescent female C57BL/6 mice (22 Mo, n = 5). Sections were Toluidine blue stained per standard protocols [60], imaged under white light excitation (200 X), combined into composites and oriented anatomically. For each composite image, the cortical bone was sub-divided and osteocytic cell lacunae present within each sub-sector (∼100 µm^2^, 384 sub-sectors) were counted for each bone and averaged across bones (young, senescent separately). This provided an estimate of the ‘average’ count of osteocytic cells present within each of 384 anatomically oriented sub-sectors. To define bone anatomy, we also imaged un-decalcified sections separately obtained from young adult (90 µm, 4 Mo, n = 5) and senescent female C57BL/6 mice (22 Mo, n = 5). The cross-section chosen for ABM implementation was representative of the average anatomy (i.e., in cortical area, thickness, moments of inertia). Upon this cross-section, each of 384 sub-sectors was ‘seeded’ with the average number of cells previously determined within the sub-sector. Cell locations within each sub-sector were then specified via a non-overlapping, random walk process. With regard to the effector cells (collectively termed ‘precursors’ in the model), per literature [61], we assumed that a single layer of precursor cells were present around the cortex and were positioned at the endocortical and periosteal surfaces (along 96 equal angle sectors). While immediately adjacent precursors were assumed to be functionally coupled, osteocyte - osteocyte coupling and osteocyte-precursor couplings were specified, per literature [62], if cell-cell separation was within a canalicular length of 50 µm (Fig 1; where ‘functional coupling’ simulates an ability to pass Ca^2+^ ions between coupled cells). Similar to literature [63], in the idealized network for young animals, the mean (± S.D.) number of functional connections per periosteal precursor was 4.0±1.6 and that for osteocytes was 7.9±2.3, and the average canalicular length was 34±12 µm. In the network for senescent animals, the mean (± S.D.) number of functional connections was 4.2±1.5 per periosteal precursor and 10.7±2.7 for the osteocyte (p<0.001 vs young), and the average canalicular length was 34±12 µm. Of note, osteocytic lacunar density was significantly increased in these aged vs young female C57BL/6 mice (940±80 vs 626±90/mm^2^; p = 0.001). However, this data is contrary to declines in lacunar density typically observed with age in healthy human bone [64], but similar to increases observed in osteoporotic vs control bones [65]. Given that, to our knowledge, there is no comparable report on age-related alterations in lacunar density in mice, the increase in lacunar density observed here may be specific to the female C57BL/6 mice at this advanced age (22 Mo) and remains to be further investigated.

### Loading induced tissue level strains at the location of a bone cell

Given the cell network topology (Fig 1), we next determined localized tissue strains at the location of each cell induced by loading of the tibia. Using a combined strain gauging and finite element modeling approach, we have previously calibrated the force, moment and tissue level strain environment that is induced at the tibia mid-shaft by loading [12], [30]. As such, for each independent loading protocol (Table S1) [12], [30], we utilized beam theory along with known force and moment boundary conditions at the tibia mid-shaft to determine tissue level strains at the location of each mechanosensory osteocytic cell over the time duration of the loading protocol.

### ABM of the Ca^2+^/NFAT pathway and relation to osteoblastic mineral apposition rates

The ABM is a biophysical model that relies on experimentally identified ‘building blocks’ to algorithmically describe how activation of the Ca^2+^/NFAT pathway by loading of bone influences mineral apposition rates induced in individual osteoblasts (Fig 1, Table S2, Protocol S1). In the model, intracellular Ca^2+^ oscillations induced in individual cells (osteocytes and precursors) at time ‘t’ during application of loading, [Ca^2+^]_i_(t), emerged via an interplay between Ca^2+^ influx due to loading induced strains at the location of the osteocytic cell (ε_i_(t)), cell-cell gap junctional exchange of Ca^2+^ ions and efflux from ER Ca^2+^ stores as follows:
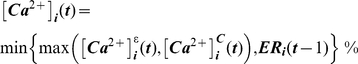
(1a)where,
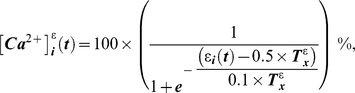
(1b)

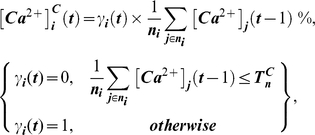
(1c)and,
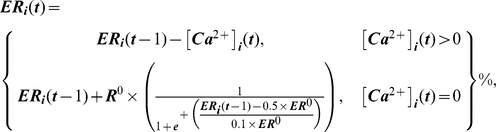
(1d)where, *T^ε^_x_* is the parameterized threshold strain magnitude required to maximize the amplitude of Ca^2+^ oscillations in mechanosensory osteocytes (normalized to 100% values), *n_i_* is the number of cells (osteocytes and/or precursor cells) functionally coupled to the i^th^ cell, *j* is an element of the cell set *n_i_*, *T^C^_n_* represents threshold ‘incoming’ Ca^2+^ ion levels required to initiate oscillations in the recipient cells (specified via preliminary simulations to be 2.5%), and *ER^0^* and *R^0^* are the parameterized *maximal* Ca^2+^ store capacity and *maximal* store recovery rates, respectively. The specific mathematical formulations for osteocyte sigmoidal dose-response relation to strain (eqn 1b) were based upon observations of Ca^2+^ oscillations induced in bone cells by mechanical stimuli [17], [38]. Further, cell-cell communication (eqn 1c) is a simple description (via averaging) of Ca^2+^ ion propagation between cells based upon reports [38], [39] and is similar to our previous descriptions [27]. The formulation for ER Ca^2+^ store depletion (eqn 1d) is similar to previously [27]. Lastly, the formulation for ER Ca^2+^ store recovery upon cellular quiescence (eqn 1d), models the action of the SERCA pump working against a Ca^2+^ ion concentration gradient and is similar to literature reports [40]–[42].

Given observations that Ca^2+^ oscillations in osteocytic networks hold potential to regulate surface osteoblastic cells [21], [22], we next consider the downstream consequences of Ca^2+^ oscillations upon NFAT transcription factor dynamics. Specifically, downstream of increased intracellular Ca^2+^ oscillations, cytoplasmic NFAT dephosphorylation and nuclear translocation in surface precursor cells was modeled to be reflective of a ‘memory’ of Ca^2+^ oscillation characteristics such as amplitude, frequency and durations, and included an implicit consideration of negative feedback elements [23], [37] as follows:
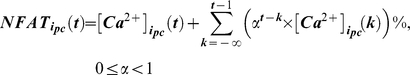
(2a)where, the parameter ‘α’ controls the amount of NFAT dephosphorylated based upon prior Ca^2+^ oscillations. The quantity of NFAT translocated into the i^th^ cell's nucleus (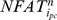) over the duration of an entire loading bout (*t_bout_*) was modeled simply as:
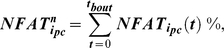
(2b)Finally, given observations that the activation of the Ca^2+^ signaling and the NFAT pathway code for highly specific cell proliferation, differentiation and apoptotic events [23], [37], [43], we assume that nuclear NFAT induced by a given loading bout controls the differentiation of precursor cells and ultimate, the relative mineral apposition rates by differentiated osteoblasts (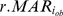) as follows:
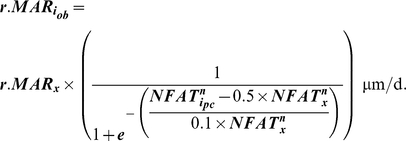
(3)where, *NFAT^n^_x_* is the maximal nuclear NFAT DNA binding capacity, and *r.MAR_x_*, the maximal relative mineral apposition rate by osteoblasts. The mathematical form (eqn 3) reflects considerations that transcriptional regulation is likely to be a threshold-driven process [44], [45].

Simulations with the ABM involved solving equations (1–3), given osteocyte level strains derived over each second of a loading bout in the context of the ABM's 6 independent parameters (i.e., specific values assigned to *T^ε^_x_*, *ER^0^*, *R^0^*, *α*, *NFAT^n^_x_*, *r.MAR_x_* prior to execution of the simulations). To initialize the ABM (at t = 0), strains induced by loading at osteocyte cell locations were 0 (i.e., ε_i_(t = 0) = 0), and the stores of all cells were assumed to be filled to capacity (i.e., ER_i_(t = 0) = ER^0^). Based upon preliminary studies, we simulated intracellular Ca^2+^ oscillations for an extra 200 seconds beyond cessation of a given loading bout to permit the unfolding of cell-cell cross talk, and NFAT dephosphorylation for an extra 100 s beyond cessation of Ca^2+^ oscillations to more fully account for prior Ca^2+^ oscillation histories.

We implemented standard dynamic histomorphometry methods to determine bone formation indices simulated by the model at the tissue level [66]. We first made the simplifying assumption that repeated loading of bone over days of the week does not adaptively alter cells (i.e., make them more/less sensitive to repeated bouts of loading). Therefore, r.MAR induced in individual osteoblasts over a loading protocol (e.g., loading for 9 bouts provided over 3 days/wk for 3 weeks) was simply determined as the average of 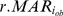 induced by each bout of loading. Second, surface referent relative mineralizing surface (r.MS/BS) at the tissue level was determined simply as the proportion of surface osteoblasts with non-zero 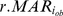. Tissue level r.MAR was then determined as the average of non-zero 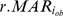 at each bone surface. Finally, tissue level relative bone formation rates (surface referent) was determined as r.BFR/BS = r.MS/BS×r.MAR at each of the surfaces. The ABM was implemented in C++ on an Apple Mac Pro (2×quad core processors, 3.0 GHz, 8 Gb RAM), and simulations were performed at 1-Hz (i.e., smallest time unit was 1 s).

### Estimating ABM parameters and statistical analysis

The ABM simulates activation of the Ca^2+^/NFAT pathway in cells and ultimately its relation to relative bone formation rates at the tissue level. However, given that the ABM is an idealized biophysical model, the parameter values must be estimated from observed rp.BFR induced by a variety of mechanical stimuli [12], [30]. We modeled the observed rp.BFR_i_ from the i^th^ loading protocol as normally distributed with population mean *μ_i_* and variance *σ_i_^2^*. The ABM with values specified for the 6 parameters **θ** = (*T^ε^_x_*, *ER^0^*, *R^0^*, *α*, *NFAT^n^_x_*, *r.MAR_x_*) at the initiation of the simulations then predicts mean values *μ_i_(θ*) = rp.BFR_i_ for each loading protocol ‘i’. Under the normal error model, the log-likelihood function for the ABM is then [67]:
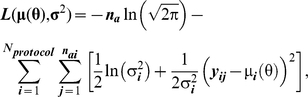
(4)where *‘y_ij_’* is the rp.BFR measured in the j^th^ animal for the i^th^ loading protocol, μ(θ) is the vector of means 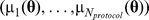 given by the ABM and **σ** ^2^ is the vector of population variances 
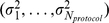
 for rp.BFR under the different loading protocols, ‘*N_protocol_*’ is the number of independent loading protocols, ‘*n_ai_*’ is the number of animals that underwent the i^th^ loading protocol, ‘*n_a_*’ is the total number of experimental observations. The maximum likelihood estimate (MLE), for the vector of means, μ(θ) and variances **σ** ^2^, is simply the set of model parameters that maximizes L(μ(θ), σ^2^). For a given vector of mean parameters μ(θ) of σ^2^, maximization with respect to the variances σ^2^ is achieved by setting:
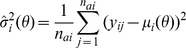
(5)However, maximization with respect to the ABM parameters θ is more challenging because the likelihood function contains local maxima. To address this, we applied the simulated annealing procedure (SA) [68], with random restarts to find the MLE for the ABM parameters. Briefly, the SA algorithm is based on annealing in metallurgy and is a probabilistic heuristic applied to a stochastic process (random walk) that seeks to determine a material configuration where energy is minimized [68]. In the context of our ABM, the SA procedure explores the 6-D model parameter space seeking to identify parameter values that would maximize the log-likelihood function L(μ(θ), σ^2^). Using this procedure, separate ABM models were calibrated to the rp.BFR data obtained from young animals subjected to 10 protocols and senescent animals subjected to 7 protocols (Table S1) [12], [30]. We denote the resulting maximum likelihood estimates of the parameter vector for each ABM by 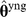 and 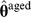 respectively (Table 2).

To analyze model simulations, we addressed the following questions (described in more detail in supporting information, Text S1): (a) Are the calibrated ABM models for young and senescent animals compatible with the observed data [12], [30]? (b) What are 95% confidence intervals for the parameters θ^yng^ and θ^aged^? c) Is there evidence against the null hypothesis that each of the ABM parameters takes value zero (i.e., simulating parameter ‘knock-outs’)? (d) Is there evidence in the observed data against the null hypothesis that the population values of the ABM parameters are the same for young and senescent animals? (e) If null hypothesis in (d) is rejected, is there evidence against the null hypotheses that specific model parameters or combination of parameters are unchanged by age? (f) Do the calibrated ABMs make predictions about the results of hypothetical interventions that would restore specific ABM parameters in senescent animals to their young values? (g) Do ABM simulations of loading induced rp.BFR, upon complete restoration of parameters significantly degraded by aging, predict data from senescent animals subject to loading supplemented with CsA? We examined these questions using a series of likelihood ratio tests (within MATLAB) and factorial ANOVAs (within SPSS) where appropriate; please see supporting information for additional details (Text S1).

### In vivo experiments

#### Ethics statement

The University of Washington Animal Care and Use committee approved all animal experiments.

As a follow-up to ABM simulations, we performed an experiment where bone formation responses were assayed in senescent mice subject to mechanical loading with/without supplementation with low-dose CsA. Specifically, the right tibia of senescent female C57BL/6 mice (22 Mo, n = 24) received mechanical loading using the noninvasive murine tibia loading device [69]. Briefly, with the mouse anesthetized with inhaled isoflurane (2%), the device grips the proximal tibia and applies controlled loads to the distal tibia in the M-L direction via a computer controlled linear force actuator. As previously [12], [30], using a combination of strain gaging, imaging via μCT and FE modeling, peak longitudinal normal strains were determined in a senescent (22 Mo) and young (4 Mo) female C57BL/6 mouse to a range of applied loads. For the in vivo experiments, loading was calibrated to induce 1700 με peak (longitudinal normal) strain for 50 cycles/d (1-Hz), three days/wk (M, W, F), for 3-wk. Animals were assigned to one of three groups and received vehicle (0.0 mg/kg, n = 8; 2% v/v DMSO, 5% v/v ethanol, 60% v/v polyethylene glycol, and 33% v/v PBS) or CsA at 0.3 mg/kg (n = 8) or 3.0 mg/kg s.c. (n = 8), 30 mins prior to each loading bout. Additionally, as a comparison, young female C57BL/6 mice (4 Mo, n = 13) received a similar loading protocol (but not CsA), with loading calibrated to induce equivalent peak periosteal longitudinal normal strains as that induced in senescent mice. All animals received Calcein labels (d 10, 19) and were killed at d 22. Bone response was determined by characterizing periosteal mineralizing surface (p.MS/BS), mineral apposition rates (p.MAR) and periosteal bone formation rate (p.BFR/BS) at the tibia mid-shaft of contralateral (left) and experimentally loaded (right) tibia via standard dynamic histomorphometry, as previously [12], [30]. Significant differences between experimental groups (p<0.05) were determined via Kruskal-Wallis non-parametric statistics with Mann-Whitney post-hoc follow-ups, and between contralateral and experimental limbs via Wilcoxon's signed rank test using SPSS statistical software.

## Supporting Information

Protocol S1 Consolidated (‘zipped’) file that includes a ‘readme’, c++ code and necessary input files for compiling and executing the code representing the developed model, and executable files compiled for both Macintosh OSX (Leopard) and windows PC based computer platforms.
(5.17 MB ZIP)

Table S1 Animal specific peak longitudinal normal strains (mean+S.E.) and rp.BFR (mean+S.E.) induced in vivo in young adult animals by 10 independent loading protocols, and in senescent animals by 7 independent loading protocols. Note that these data represent mean values after removal of outliers (mean ± 2×S.D.). This resulted in the removal of 1 data point in young adult animals (from n = 70) and of 4 data points in senescent animals (from n = 56).
(0.07 MB DOC)

Table S2 Definitions for notations and symbols used in manuscript.
(0.09 MB DOC)

Text S1 Methods describing previous data set used to calibrate model and statistical analysis.
(0.12 MB DOC)
