# Supplementary material for: Rescuing Loading Induced Bone Formation at Senescence
Source: PLoS Comput Biol. 2010 Sep 9;6(9):e1000924. doi: 10.1371/journal.pcbi.1000924 (PMC2936512; doi:10.1371/journal.pcbi.1000924)
Supplement: Table S1 — Animal specific peak longitudinal normal strains (mean+S.E.) and rp.BFR (mean+S.E.) induced in vivo in young adult animals by 10 independent loading protocols, and in senescent animals by 7 independent loading protocols. Note that these data represent mean values after removal of outliers (mean ± 2×S.D.). This resulted in the removal of 1 data point in young adult animals (from n = 70) and of 4 data points in senescent animals (from n = 56). (0.07 MB DOC) [file pcbi.1000924.s002.doc]

Table S1: Loading regimens, animal specific strains and rp.BFR induced in mice.

|  | Loading Regimen | | | Induced Strain (me) | rp.BFR (μm^3^/μm^2^/d) |
| --- | --- | --- | --- | --- | --- |
|  | Strain (me) | Cycles/d | Rest (s) |  |  |
| Young Adult (4 Mo) | 1000 | 50 | 0 | 1022 + 40 | 0.029 + 0.011 |
|  | 1250 | 50 | 0 | 1226 + 26 | 0.100 + 0.032 |
|  | 1600 | 50 | 0 | 1567 + 49 | 0.215 + 0.059 |
|  | 1000 | 50 | 10 | 1003 + 37 | 0.085 + 0.032 |
|  | 1250 | 50 | 10 | 1275 + 40 | 0.168 + 0.050 |
|  | 1600 | 50 | 10 | 1640 + 48 | 0.377 + 0.084 |
|  | 1250 | 10 | 0 | 1268 + 49 | 0.042 + 0.026 |
|  | 1250 | 250 | 0 | 1249 + 48 | 0.154 + 0.054 |
|  | 1250 | 10 | 10 | 1266 + 38 | 0.133 + 0.038 |
|  | 1250 | 250 | 10 | 1249 + 40 | 0.414 + 0.067 |
| Senescent (22 Mo) | 1200 | 50 | 0 | 1223 + 61 | 0.023 + 0.012 |
|  | 1200 | 500 | 0 | 1193 + 58 | 0.065 + 0.025 |
|  | 2400 | 50 | 0 | 2420 + 103 | 0.097 + 0.013 |
|  | 1200 | 50 | 10 | 1262 + 70 | 0.104 + 0.024 |
|  | 2400 | 50 | 10 | 2242 + 143 | 0.104 + 0.015 |
|  | 2400 | 50 | 20 | 2558 + 165 | 0.086 + 0.025 |
|  | 2400 | 250 | 20 | 2688 + 179 | 0.156 + 0.032 |
