# Supplementary material for: Rescuing Loading Induced Bone Formation at Senescence
Source: PLoS Comput Biol. 2010 Sep 9;6(9):e1000924. doi: 10.1371/journal.pcbi.1000924 (PMC2936512; doi:10.1371/journal.pcbi.1000924)
Supplement: Table S2 — Definitions for notations and symbols used in manuscript. (0.09 MB DOC) [file pcbi.1000924.s003.doc]

Table S2: Notations used in manuscript and their definitions

| Notation | Definitions |
| --- | --- |
|  | Strain at the location of the i^th^ mechanosensory osteocyte at time ‘t’ |
|  | Ca^2+^ oscillation induced in cell ‘i’ at time ‘t’ |
|  | Ca^2+^ oscillation induced in i^th^ cell by strain $e_{i}(t)$ |
|  | Ca^2+^ oscillation induced in i^th^ cell by cell-cell signaling |
|  | Amount of Ca^2+^ within ER store of i^th^ cell at time ‘t’ |
|  | **Model parameter** that defines the threshold strain magnitude that maximizes Ca^2+^ oscillation amplitudes |
|  | **Model parameter** defining the maximal ER Ca^2+^ store capacity |
|  | **Model parameter** defining the maximal ER Ca^2+^ store recovery rate |
|  | Dephosphorylated NFAT accumulated in i^th^ precursor cell at time ‘t’ |
|  | **Model parameter** that controls the amount of NFAT dephosphorylated based upon prior Ca^2+^ oscillations |
|  | Time duration of a given loading bout |
|  | Dephosphorylated NFAT translocated to the i^th^ precursor cell’s nucleus |
|  | **Model parameter** defining the maximal nuclear NFAT DNA binding capacity |
|  | **Model parameter** defining the maximal relative mineral apposition rate in osteoblasts |
|  | Relative mineral apposition rate induced in the i^th^ osteoblast |
|  | Tissue level relative mineral apposition rate |
|  | Tissue level surface referent relative mineralizing surface |
|  | Tissue level surface referent relative bone formation rate |
|  | Tissue level surface referent relative periosteal bone formation rate |
|  | Vector of model parameters ($T_{x}^{e}$,$\text{ER}^{0}$,$R^{0}$,$\alpha$,,$r.\text{MAR}_{x}$) |
|  | Mean rp.BFR value simulated by the model for the i^th^ loading protocol |
|  | Model variance in simulating rp.BFR values for the i^th^ loading protocol |
|  | Log-likelihood function for the model, given the vector of means, *μ(θ)*, and variances, σ*^2^* |
