# Supplementary material for: Rescuing Loading Induced Bone Formation at Senescence
Source: PLoS Comput Biol. 2010 Sep 9;6(9):e1000924. doi: 10.1371/journal.pcbi.1000924 (PMC2936512; doi:10.1371/journal.pcbi.1000924)
Supplement: Text S1 — Methods describing previous data set used to calibrate model and statistical analysis. (0.12 MB DOC) [file pcbi.1000924.s004.doc]

**Supporting Information - Methods**

**Experimental data for model calibration**

In two previous studies, we have explored the response of bone in young adult (4 Mo, n = 70) and senescent female C57BL/6 mice (22 Mo, n = 56) to a variety of mechanical stimuli. Briefly, the right tibiae of young adult female C57BL/6J mice (4 Mo, n = 70) were subject to mechanical loading (‘cantilever’ bending in the M-L direction), 3 d/wk for 3-wk using the non-invasive murine tibia loading device [30]. Animals were randomly assigned to groups (n = 7/grp) in which cyclic or 10-s rest-inserted loading induced a range of peak normal strains at the tibia mid-shaft over a fixed cycle number (1000 με, 1250 με, or 1600 με for 50 cycles/d), or varied cycle numbers at a fixed strain (10, 50, or 250 cycles/d at 1250 με). Animals received calcein labeling (d 10, 19), were killed on d 22 and mid-shaft cross-sections (90 μm) were imaged via fluorescence microscopy. Bone formation rates were determined using dynamic histomorphometry, and animal specific induced strains were determined via beam theory. Finally, relative periosteal bone formation rates (rp.BFR) were determined as the bone formation rates in the loaded – contralateral tibiae (Table S1).

In studies with senescent animals [12], groups of aged female C57BL/6 mice (22 Mo, n = 56) underwent mechanical loading of the right tibia 5 days/wk for 2-weeks, using the non-invasive murine tibia loading device. Animals were randomly assigned to receive loading calibrated to induce 1200 με peak longitudinal normal strain at the tibia mid-shaft over 50 cycles/d (n = 8) or 500 c/d (n = 7; previously unpublished data) applied as a cyclical waveform (1-Hz) or over 50 cycles/d as a 10 s rest-inserted waveform (n = 9). Additional animals received loading calibrated to induce 2400 με peak strain applied cyclically (1-Hz, n = 9), with a 10 s (n = 9) or 20 s rest inserted between each of 50 load cycles (n = 7), or with 10 s rest inserted between each of 250 load cycles (n = 7). Animals received calcein labeling (d 3, 12) and upon sacrifice (d 15), loading induced animal specific strains and rp.BFR were determined as above for young adult animals (Table S1).

**Statistical analysis of ABM simulations**

Upon model calibration, we performed statistical analyses to address the following questions using MATLAB and SPSS statistical analysis software:

1. Are the calibrated ABM models for young and senescent animals compatible with the observed data?
2. What are 95% confidence intervals for the parameters $q^{\text{yng}}$ and $q^{\text{aged}}$?
3. Is there evidence against the null hypothesis that each of the ABM parameters takes value zero?
4. Is there evidence in the observed data against the null hypothesis that the population values of the ABM parameters are the same for young and senescent animals, i.e. *H*_0_ against the alternative: *H*_1_ ?
5. If the null hypothesis in (d) is rejected, can we say anything about whether there is evidence against the null hypotheses that specific model parameters or parameter combinations are unchanged by aging?
6. Do the calibrated ABMs make predictions about the results of hypothetical interventions that would restore specific ABM parameters in senescent animals to their young values?
7. Do ABM simulations of loading induced rp.BFR, upon complete restoration of parameters significantly degraded by aging, predict data from senescent animals subject to loading supplemented with CsA?

We address questions (a)-(e) via likelihood ratio tests, and (f) and (g) via ANOVA.

Post estimation (via maximizing the likelihood) of the two vectors of ABM parameters ($\hat{q}^{\text{yng}}$ and $\hat{q}^{\text{aged}}$) we used the likelihood ratio test (LRT) to explore parameter accuracy, confidence intervals, redundancy as well as to determine whether aging significantly alters the modeled parameters. Briefly, the LRT determines the ratio of the maximum value attained by the likelihood (L_1_) using an unconstrained model to the maximum value attained by likelihood (L_0_) under a further constrained model that represents the ‘null-hypothesis’. Under the assumption that the null model is true, then

…(s1)

will follow a chi-squared distribution with degrees of freedom given by the number of parameters constrained by null hypothesis, say *k*. Consequently, large values of LRT represent evidence against the null hypothesis. We may compute (asymptotic) p-values by computing P($c_{k}^{2}$>LRT), where $c_{k}^{2}$ is a chi-square random variable with degrees of freedom *k*, and LRT is the observed value of the likelihood ratio statistic. A conventional hypothesis test can then be performed by comparing the p-value to 0.05. We briefly describe the specific statistical tests that were performed for each of the questions outlined previously.

*(a) Are the fitted ABM models for young and senescent animals compatible with the observed data?* We performed a LRT comparing the value of the likelihood (L_0_) attained by a null model under which separate ABMs were used to model the observed bone formation rates for each age group (using 6 + 6 = 12 parameters in total), to an unconstrained model (L_1_) which allowed a separate mean for each protocol and age group, giving 17 parameters in total. Note that the maximum likelihood estimates under the latter model are given by:

; . …(s2)

where y_ij_ is rp.BFR data observed in the j^th^ animal subject to the i^th^ loading protocol, and n_ai_ is the number of animals enrolled within the i^th^ loading protocol. Under the null hypothesis that the two ABM models describe the true distribution of bone formation rates for young adult and senescent animals (Table S1) [12, 30], asymptotically the LRT statistic will follow a χ^2^ distribution with 17 – 12 = 5 d.f. When calibrating the ABM we make the simplifying assumption that the strain experienced by each animal within a protocol is the same. We checked this approximation by implementing animal specific loading induced strains as inputs and by comparing the animal specific rp.BFR (Table S1) [12, 30], with ABM simulations for young and aged animals (using the maximum likelihood estimates $\hat{q}^{\text{yng}}$ and $\hat{q}^{\text{aged}}$**)**, via a factorial ANOVA test.

(b) *What are 95% confidence intervals for the ML estimates $q^{\text{yng}}$ and* $q^{\text{aged}}$***?***

We constructed 95% confidence intervals for each of the 6 ABM parameters for young and senescent ABMs by inverting LRTs. Thus for each parameter , grp ∈ [yng, aged], i ∈ [1,…,6], we determined the range such that for any $q_{0}$∈ the null hypothesis =$q_{0}$ is not rejected at significance level 0.05, against the alternative , by the LRT. This is a χ^2^ test on 1 d.f. For example, to determine the 95% confidence interval for the model parameter T^ε^_x_[yng], we constructed a null model where T^ε^_x_[yng] = C1 (a constant and pre-specified value) but where the remaining 5 parameters (i.e., ER^0^[yng], R^0^[yng], α[yng], NFAT^n^_x_[yng], r.MAR_x_[yng]) were unconstrained. The null model’s unconstrained parameters (values) were then estimated by finding the parameter values for which the predictions of the ABM provided the best fit (i.e. highest likelihood, see eqn 4) to the in vivo rp.BFR data in young mice (Table S1). The values of the parameters that maximize the likelihood were obtained via simulated annealing. The maximum likelihood value for the null model was then contrasted with the maximum likelihood value for an alternate model, in which T^ε^_x_[yng] is free to vary, and not fixed to C1 (using the optima, young parameter values in Table 2) via the LRT (on 6-5 = 1 d.f.). Thus, if the null model was accepted (p ≥ 0.05), the value C1 is within the confidence interval for the parameter T^ε^_x_[yng]; otherwise, it would lie outside the 95% confidence interval. Since performing LRTs is computationally intensive (it requires optimization of all unconstrained ABM parameters for each value ‘C’ for each ), the endpoints of the confidence interval were estimated via linear interpolation from a fixed grid of 25 values on either side of the MLE, $\hat{q}_{i}^{\text{grp}}$ (Table 2).

(c) *Is there evidence against the null hypothesis that each of the ABM parameters takes value zero?*

If the null hypothesis in (a) is not rejected, and the ABM models for young and aged bone-formation rates are compatible with the observed data, it is natural to ask whether the model of the Ca^2+^/NFAT pathway may be simplified further. Thus for each of the ABM parameters, we tested the specific null hypothesis (L_0_) that the value of the parameter was zero (by maximizing the likelihood for the null model) against the alternative that it was non-zero (L_1_). These simulations, performed for the case of data from young animals, also served as in silico functional ‘knockout’ experiments of the model parameters. Under the null hypothesis that the population value was zero, the LRT will follow (asymptotically) a χ^2^ distribution with 6 – 5 = 1 d.f. If the null hypothesis was significant, we then implemented animal specific loading induced strains as inputs and compared the animal specific rp.BFR (Table S1) [12, 30], with ABM simulations (at the ‘intact’ vs ‘knockout’ MLEs) using a factorial ANOVA test and Tukey's HSD post-hoc follow-ups.

(d) *Is there evidence in the observed data against the null hypothesis that the population values of the ABM parameters are the same for young and senescent animals, i.e. H_0_ against the alternative: H_1_ ?*

We next tested whether the observed bone formation rates provided evidence against the null hypothesis that aging does not alter the modeled mechanisms representing the Ca^2+^/NFAT pathway and its relation to bone matrix secretion by osteoblasts (analogous to examining the ‘main model’ for significance in a multi-factorial ANOVA). For this test, the null model (L_0_) constrained the ABM parameters for young and old to be equal, and thus contained 6 parameters that were re-estimated by maximizing the likelihood for the null model via simulated annealing. The alternative model (L_1_) was the (null) model used in (a), which allowed separate parameters for young ($q^{\text{yng}}$**) **and aged ($q^{\text{aged}}$**)**. Thus under the null hypothesis, the LRT will follow (asymptotically) a χ^2^ distribution on 6 d.f.

(e) *If the null hypothesis in (d) is rejected, can we say anything about whether there is evidence against the null hypotheses that specific model parameters or parameter combinations are unchanged by aging?*

Given the results of the previous test, we next explored whether the observed rp.BFR data provided evidence against particular null hypotheses that specific ABM parameters corresponding to mechanisms of the Ca^2+^/NFAT pathway were unchanged by aging (akin to post-hoc analysis following an ANOVA). In these tests, the null model (L_0_) contained 11 parameters (θ_i_^yng^ = θ_i_^aged^, plus the 10 parameters θ_k_^yng^, θ_k_^aged^ for ki) while the alternative model (L_1_) was the previously estimated, 12 parameter ABM (with the MLE in Table 2). For example, to construct the null model when age-related alterations in a single parameter is being considered (say T^ε^_x_[yng] = T^ε^_x_[aged]?), the original 12 parameter model (6 for young and 6 for old) is restricted so that it now only contains 11 unconstrained parameters (i.e., T^ε^_x_[yng] = T^ε^_x_[aged], ER^0^[yng], R^0^[yng], α[yng], NFAT^n^_x_[yng], r.MAR_x_[yng], and ER^0^[aged], R^0^[aged], α[aged], NFAT^n^_x_[aged], r.MAR_x_[aged]). The 11 parameters of each null model were then estimated via the method of maximum likelihood to find the values for which the ABM produces predictions that provide the best fit (i.e. highest value of the likelihood; see Eqn 4) for the in vivo rp.BFR data in young [30] and old mice [12]. The values of these 11 parameters were found via simulated annealing. The maximum value of the likelihood attained by each such null model were then contrasted with the maximum likelihood value for the alternate model (from (a)), which does not constrain θ_i_^yng^ = θ_i_^aged^, via a likelihood ratio test (LRT) (on 12-11 = 1 d.f.). The LRT compares the observed difference between the two maximized values of the likelihood, under the Null and Alternate models, with the distribution of such differences that would be expected if the Null hypothesis model were true. The LRT then indicates whether a given parameter is altered with age (p < 0.05, rejection of the null) or not altered with age (p ≥ 0.05, failure to reject the null). Based on our reading of the bone adaptation literature, we similarly examined null hypotheses in which subsets of parameters, when considered synchronously together, were not altered with aging.

(f) *Do the calibrated ABMs make predictions about the results of hypothetical interventions that would restore specific ABM parameters in senescent animals to their young values?*

For the ABM parameters significantly altered by aging, we explored the benefit of synchronously restoring deficits in parameters upon loading induced bone formation rates. For this, we initialized the model by restoring all parameters significantly altered by aging to their ‘young’ optima parameter values, while retaining the remaining parameters at their aged optima values (Table 2). We then simulated rp.BFR induced by the 7-independent protocols in senescent animals (using animal specific strains) (Table S1) [12], and contrasted model predictions with rp.BFR simulated by the model when all parameters were assigned their aged optima values. The significance of benefits derived by restoring age-related deficits was determined using factorial ANOVA and Tukey's HSD post-hoc follow-ups.

(g) *Do ABM simulations of loading induced rp.BFR, upon complete restoration of parameters significantly degraded by aging, predict data from senescent animals subject to loading supplemented with CsA?*

To examine the validity of model predictions, we simulated rp.BFR induced in our in vivo experiments (Fig 5) when senescent animals were subject to loading calibrated to induce 1700 με peak strain without or with CsA supplements (0.3. mg/Kg and 3.0 mg/Kg) and in young animals exposed to loading calibrated to induce equivalent peak periosteal strains as senescent animals. As the model currently does not have an explicit ability to simulate CsA dosages, we assumed that either of the low-dose CsA supplements (0.3 or 3.0 mg/Kg) would completely restore aged parameters α, and NFAT^n^_x_ to their young values. Given animal specific strains in the experimental groups determined via beam theory, tissue level rp.BFR was simulated for senescent animals subject to loading without/or with complete restoration of α, and NFAT^n^_x_ and for young animals. Model simulations were then contrasted and validated against experimental data via factorial ANOVA.
