# Supplementary material for: Rescuing Loading Induced Bone Formation at Senescence
Source: PLoS Comput Biol. 2010 Sep 9;6(9):e1000924. doi: 10.1371/journal.pcbi.1000924 (PMC2936512; doi:10.1371/journal.pcbi.1000924)
Supplement: Protocol S1 — Consolidated (‘zipped’) file that includes a ‘readme’, c++ code and necessary input files for compiling and executing the code representing the developed model, and executable files compiled for both Macintosh OSX (Leopard) and windows PC based computer platforms. (5.17 MB ZIP) [file pcbi.1000924.s001.zip › ABMV_PLoS/readme.doc]

(I)Source Files:

CPP files:

(1) Agent.cpp

(2) EvalObjFun.cpp

(3) Input.cpp

(4) Iterate.cpp

(5) Output.cpp

(6) main.cpp

Header Files:

(7) Agent.h

(8) EvalObjFun.h

(9) Input.h

(10) Iterate.h

(11) Output.h

See individual files for their descriptions.

(II) Input Files:

(1) ParaOptima.txt contains parameters (in order): Young (TeMax, Rt, CaStore0, Decay, nucNFATMax, MARmax) and Aged (TeMax, Rt, CaStore0, Decay, nucNFATMax, MARmax), where:

TeMax is the parameterized threshold strain magnitude required to maximize the amplitude of Ca^2+^ oscillations in mechanosensory osteocytes (referred to as ‘T^ε^_x_’ in the manuscript).

Rt is the maximal store recovery rate (referred to as ‘R^0^’ in the manuscript).

CaStore0 is the parameterized *maximal* Ca^2+^ store capacity (referred to as ‘ER^0^’ in the manuscript).

Decay is a parameter controlling the amount of NFAT dephosphorylated based on prior Ca2+ oscillation histories (referred to as ‘α’ in the manuscript).

nucNFATMax is the maximal nuclear NFAT DNA binding capacity (referred to as ‘NFAT^n^_x_’ in the manuscript).

MARmax the maximal relative mineral apposition rate by osteoblasts (referred to as ‘r.MAR_x_’ in the manuscript).

(2) precursor_aged.txt contains coordinates of precursor cells in aged tibia mid-diaphysis: x(first column) and y(second column); Row-wise first half endocortical and second half periosteal.

(3) precursor_young.txt contains coordinates of precursor cells in young tibia mid-diaphysis: x(first column) and y(second column); Row-wise first half endocortical and second half periosteal.

(4) ocytes_aged.txt contains coordinates of osteocytes in aged tibia mid-diaphysis: x(first column) and y(second column).

(5) ocytes_young.txt contains coordinates of osteocytes in young tibia mid-diaphysis: x(first column) and y(second column).

(6) prot_aged.txt contains loading protocol details for the 7 protocols implemented in senescent mice (rows). Each row has the following 4 data in order: Max Strain (με), No. of cycles, rest duration (s), number of load cycles after with rest is inserted.

(7) prot_young.txt contains loading protocol details for the 10 protocols implemented in young mice (rows). Each row has the following 4 data in order: Max Strain (με), No. of cycles, rest duration (s), number of load cycles after with rest is inserted.

(III) Output Files:

(a) Ca2+

The Ca2+ amplitude output file for an aged mouse for the m-th protocol is Ca_Aged_Prot_m.txt , where m = 1 to 7.

The Ca2+ amplitude output file for a young mouse for the n-th protocol is Ca_Young_Prot_n.txt , where n = 1 to 10.

The data (for each periosteal precursor at each time step) are arranged as Time(s) in Rows; Precursor No. in Columns. Note periosteal precursor locations are as specified, in order, in the second half of precursor_aged.txt and precursor_young.txt files for aged and young simulations, respectively.

The first column contains time; the second column contains the corresponding Ca2+ amplitude for the 1^st^ periosteal precursor; the third column for the 2^nd^ periosteal precursor and so on.

(b) NFAT

The NFAT output file for an aged mouse for the m-th protocol is NFAT_Aged_Prot_m.txt , where m = 1 to 7.

The NFAT output file for a young mouse for the n-th protocol is NFAT_Young_Prot_n.txt , where n = 1 to 10.

The data (for each periosteal precursor at each time step) are arranged as Time(s) in Rows; Precursor No. in Columns.

The first column contains time; the second column contains the corresponding NFAT values for the 1^st^ periosteal precursor; the third column for the 2^nd^ periosteal precursor and so on.

(c) rp.MAR

The periosteal r.MAR output file for an aged mouse is rp.MAR_Aged_.txt .

The periosteal r.MAR output file for a young mouse is rp.MAR_Young_.txt .

The data (for each periosteal osteoblast for each protocol) are arranged as Protocol No. in Rows; Osteoblast No. in Columns.

The first column contains Protocol No.; the second column contains the corresponding rp.MAR values for the 1^st^ periosteal osteoblast; the third column for the 2^nd^ periosteal osteoblast and so on.

(d) rp.BFR

The rp.BFR output file for an aged mouse is rp.BFR_Aged_.txt .

The rp.BFR output file for a young mouse is rp.BFR_Young_.txt .

The first column contains Protocol No.; the second column contains the corresponding tissue level rp.BFR.

(IV) Executable Files:

(1) MAC OS X (Leopard)

(i) ABMV

(2) Windows XP (32-bit)

(i) vcredist_x86.exe ( this is Microsoft Visual C++ 2010 Redistributable Package downloaded from <http://www.microsoft.com/downloads/details.aspx?familyid=A7B7A05E-6DE6-4D3A-A423-37BF0912DB84&displaylang=en> . Please install this file before running ABMV.exe if Visual C++ is not already installed on the target computer.)

(ii) ABMV.exe

(V) Running the Program

Place the program executable files and all the input files in the same folder. Click on ABMV or on the command prompt, move to that folder and type the following:

"./ABMV" for MAC OSX and "ABMV.exe" for Windows XP.

**For Windows XP, please make sure that either vcredist_x86.exe or Visual C++ is installed on the target computer before running the program.**

The program should take a few seconds to write the output files.
